# Obesity and diabetes mellitus association in rural community of Katana, South Kivu, in Eastern Democratic Republic of Congo: Bukavu Observ Cohort Study Results

**DOI:** 10.1186/s12902-016-0143-5

**Published:** 2016-11-11

**Authors:** Philippe Bianga Katchunga, Justin Cikomola, Christian Tshongo, Arsene Baleke, David Kaishusha, Patrick Mirindi, Théodore Tamburhe, Yves Kluyskens, Antoine Sadiki, Socrate Bwanamudogo, Zacharie Kashongwe, Marc Twagirumukiza

**Affiliations:** 1 Observatory NCDs VLIR-UOS/UCB, Faculty of Medicine of the Catholic University of Bukavu, Bukavu, South-Kivu Province Democratic Republic of the Congo; 2 Division of Endocrinology and Diabetology, Department of Internal Medicine, Reference Provincial General Hospital of Bukavu, Bukavu, South-Kivu Province Democratic Republic of the Congo; 3 Regional School of Public Health (ERSP), Faculty of Medicine of the Catholic University of Bukavu, Bukavu, South-Kivu Province Democratic Republic of the Congo; 4 Institut de technique médicale, hôpital général de référence de Katana, Katana, Sud-Kivu Democratic Republic of the Congo; 5 Department of Pharmacology, Faculty of Medicine and Health sciences, Ghent University, Ghent, Belgium; 6 Department of Clinical Biology, Reference Provincial General Hospital of Bukavu, Bukavu, South-Kivu Province Democratic Republic of the Congo

**Keywords:** Obesity, Diabetes mellitus, Democratic Republic of the Congo, Sub-Saharan Africa

## Abstract

**Background:**

Factual data exploring the relationship between obesity and diabetes mellitus prevalence from rural areas of sub-Saharan Africa remain scattered and are unreliable. To address this scarceness, this work reports population study data describing the relationship between the obesity and the diabetes mellitus in the general population of the rural area of Katana (South Kivu in the Democratic Republic of the Congo).

**Methods:**

A cohort of three thousand, nine hundred, and sixty-two (3962) adults (>15 years old) were followed between 2012 and 2015 (or 4105 person-years during the observation period), and data were collected using the locally adjusted World Health Organization’s (WHO) STEPwise approach to Surveillance (STEPS) methodology. The hazard ratio for progression of obesity was calculated. The association between diabetes mellitus and obesity was analyzed with logistic regression.

**Results:**

The diabetes mellitus prevalence was 2.8 % versus 3.5 % for obese participants and 7.2 % for those with metabolic syndrome, respectively. Within the diabetes group, 26.9 % had above-normal waist circumference and only 9.8 % were obese. During the median follow-up period of 2 years, the incidence of obesity was 535/100,000 person-years. During the follow-up, the prevalence of abdominal obesity significantly increased by 23 % (*p* <0.0001), whereas the increased prevalence of general obesity (7.8 %) was not significant (*p* = 0.53). Finally, diabetes mellitus was independently associated with age, waist circumference, and blood pressure but not body mass index.

**Conclusion:**

This study confirms an association between diabetes mellitus and abdominal obesity but not with general obesity. On the other hand, the rapid increase in abdominal obesity prevalence in this rural area population within the follow-up period calls for the urgent promoting of preventive lifestyle measures.

## Background

Diabetes mellitus (DM) is nowadays known as a major public health problem worldwide because of its continuously increasing prevalence and the risk of associated cardiovascular and renal diseases. Indeed, 382 million people were estimated to have DM in 2013; the number of people with DM is predicted to reach 592 million by 2035 [1]. In contrast, this disease affected only 30 and 157 million people in 1985 and 2000, respectively [2].

According to data from occidental settings and most developed areas in low- and middle- income countries, the increase of diabetic patient’s number is mainly due to the increasing figures of obese people and aging of the general population [3–5].

The association between DM and obesity with or without other cardiovascular risk factors is known as the ‘diabesity’ [6]. It also has an insulin resistance pattern, which is clinically determined by the criteria of the metabolic syndrome (MS) [7].

The prevalence of ‘diabesity’ is rapidly increasing in Africa following the worldwide trend [8, 9]. This progress has been largely linked to changes in lifestyles of the inhabitants, especially those living in urban areas [8–10]. However, in rural Africa, the context is quite different because no substantial changes are seen. Field and manual work (agricultural, rural daily activities, etc.) are active, physical exercises that may contribute to the reduced prevalence of obesity. However, it is not well-documented whether this assumed low prevalence of obesity in rural areas matches with DM prevalence.

Furthermore, in the sub-Saharan African setting the DM characteristic is specific. Considered atypical, it is mainly described with a low frequency of insulin resistance [11–13]. Based on the current scarce data, it is even speculated that the frequency of atypical diabetes would be more important in rural areas [10].

In the Democratic Republic of the Congo (DRC), data on “diabesity” in rural areas are very rare and screening for the disease in this area is very low [10].

In 2012, an observatory to monitor the development of non-communicable diseases and risk factors, including arterial hypertension (AHT) and DM, was established in South Kivu in eastern DRC. The ultimate goal of this observatory is to implement control strategies against these risk factors [14].

The present work presents the partial results about the relationship between DM and obesity from one site of the cohort study.

## Methods

### Study population

An observatory center that continuously monitors and collects factual data regarding the prevalence of non-communicable diseases including DM, AHT, and other cardiovascular risk factors has been established at the Catholic University of Bukavu Medical School in collaboration with Ghent University in Belgium (Bukavu ObServ Study).

The rural survey was conducted in the village of Ciranga [estimated 8765 inhabitants] in Katana, a rural zone 45 km from Bukavu. All adult subjects of both genders aged 15 years or older (approximately 50 % of the population) and living in the site were asked to participate. Their verbal informed consent was obtained. At the end of the first cycle of the survey (2012–2013), 1601 households in rural areas were visited and 4345 adults were found. A total of 108 (2.4 %) subjects declined to participate in the study. The acceptance rate was 97.6 %.

In 2015 (end of the second cycle), 3962 subjects were found. Of these cases, 2088 were old cases (49.2 %) including 26 deaths and 1874 were newly recruited. These constitute the 3936 participants of the study.

### Data collection

#### Baseline examination (2012)

Data collection was based on a systematic sampling method and included all residents in a selected administrative area at the time of data collection.

Teams of trained investigators visited the participants at their home and collected data using a standardized questionnaire and physical measurements.

The questionnaire used was adapted from the World Health Organization’s STEP (WHO-STEP) questionnaire for the local context [15]. It included questions on individual data, family history, lifestyle habits such as smoking and alcohol, weekly physical activity, dietary habits, medical history of the subjects, the notion of cardiovascular risk factors, and management of chronic medications.

Then, body weight was measured to the nearest 100 gr while the subject was dressed only in light-weight clothing using an electronic scale (Tanita Digital Bathroom Scale HD-325®); height was determined with a SECA mesband 206 cm® and with a tape measure; waist circumference (WC) was measured between the 12th rib to the iliac crest at the end of expiration to nearest 0.5 centimeter.

Finally, blood pressure and pulse rate were determined using an electronic device (OMRON Hem 7001E®) These measurements were taken on the right arm, which was supported at heart level, after the subject had been seated and relaxed for at least 5 min.

The average of three consecutive measurements was used for analysis. Sphygmomanometers were calibrated monthly with a mercury device and any difference of 4 mmHg or more disqualified use of the device. Bedridden persons and/or those with edematous syndrome and pregnant women were excluded from the measurements.

#### Cohort demographic data monitoring

Because in the selected area there is no reliable and continuous census data available, the Bukavu ObServ study included yearly census-like surveys, known as the Demographic Surveillance System (DSS). The goal of those surveys is to monitor the demographic dynamics of the study population and to document all changes in the denominator figures used for incidence and prevalence calculations. This survey was done each year (since 2012–the baseline) and is based on a short questionnaire that asks questions about the household baseline composition and in-and-out dynamics (i.e., births, immigrations, emigrations, and deaths). The questionnaire included also questions related to socio-economic components and their changes over time.

#### Follow-up data collection

In 2015, investigators revisited all households in the same administrative areas that were visited during the baseline survey and re-examined the present subjects using the same methodology as in baseline surveys. For this 2015 phase, anthropometric parameters (weight, height, waist circumference, and neck circumference) were taken under the same conditions as in baseline surveys.

Then, capillary blood from a finger puncture was immediately analyzed for glucose concentration by the glucose oxidase method using a portable electronic blood glucose monitor (Codefree®) with commercially available strips. This method was previously calibrated in a hospital setting with a laboratory-based method.

When fasting glucose (last meal > 8 h) or postprandial blood glucose (last meal <8 h) were ≥ 126 mg/dl and ≥ 140 mg/dl, respectively, a fasting control test was performed the next day.

Finally, 4 mL of blood were obtained from an antecubital venopuncture for lipid, creatinine, and uric acid determinations by standard methods of spectrophotometry. Similarly, qualitative proteinuria by dipstick was assessed.

### Study outcomes

DM was defined as fasting glucose ≥ 126 mg/dl and/or taking hypoglycemic medication; prediabetes was defined as a fasting glucose between 100 and 125 mg/dl. High blood pressure was defined as systolic blood pressure (SBP) ≥ 140 mmHg and/or diastolic blood pressure (DBP) ≥ 90 mmHg and/or taking antihypertensive medications [16]. Body mass index (BMI) scores less than 18.5 kg/m^2^, between 18.5 and 24.9 kg/m^2^, between 25 and 29.9 kg/m^2^ and ≥ 30 kg/m^2^ were defined as malnourished, normal weight, overweight, and obese, respectively [17]. Abdominal obesity was defined by a WC ≥ 94 cm for both men and women. WC ≥ 94 cm provided the same rate of abdominal obesity as reported in our previous study [18] and in other studies [19, 20]. MS has been defined as the presence of three or more of the following five risk factors: 1) abdominal obesity (WC ≥ 95 cm in men and in women); 2) hypertriglyceridemia (triglycerides (TG) ≥ 150 mg/dL or use of medications to lower triglycerides); 3) decreased high-density lipoprotein cholesterol HDL-C (<40 mg/dL in men and <50 mg/dL in women or use of medication to increase HDL-C); 4) high blood pressure (systolic blood pressure ≥130 mmHg and/or diastolic blood pressure ≥85 mmHg and/or use of antihypertensive medication); 5) high fasting plasma glucose ≥100 mg/dL or use of hypoglycemic medication. Intense professional activity was work which required a significant increase in breathing and heart rate. High intensity leisure activities such as sports were categorized as “intense activity”. A moderate usual physical activity such as walking for at least 10 min or displacement by bicycle was categorized as “moderate activity”.

### Statistical analysis

The software Epi-Info® 2008 version 3.5.1 (Centers for Disease Control and Prevention, Atlanta, GA, USA) and MedCalc® Version V12.4.0 (MedCalc Software, Ostend, Belgium) were used for statistical analysis. Data are presented as appropriate, by the median (interquartile range) or the relative frequency in percent. The incidence of obesity in each group was estimated using the Kaplan–Meier product-limit estimator, and groups were compared using the log-rank test. The rate of progression to obesity was calculated in each group by dividing the number of individuals with obesity by the number of person-years of observation. Hazard ratios and 95 % confidence intervals (CIs) for progression to obesity were analyzed using the Cox proportional hazards model.

The occurrence of DM and MS according to candidate risk factors was studied by multiple logistic regressions.

A probability (*p*) <0.05 was considered statistically significant.

## Results

### General characteristics of the study population

Table 1 shows the general characteristics of the study population. Of the 3936 subjects, 2213 (56.2 %) were female and 1723 (43.7 %) were men.

**Table 1:** General characteristics of the study population

	Total group *n* = 3.962 (100 %)	malnourished *n* = 269 (6.8 %)	Normal weight *n* = 3.003 (75.8 %)	Overweight *n* = 551 (13.9 %)	Obese *n* = 139 (3.5 %)	*p*
Age (years)	34.0 (22.0 to 50.0)	39.5 (20.0 to 63.5)	32.0 (22.0 to 48.0)	36.0 (25.0 to 45.0)	43.0 (31.0 to 52.0)	<0.0001
BMI (kg/m^2^)	22.2 (20.5 to 24.1)	17.7 (17.0 to 18.1)	21.8 (20.5 to 23.1)	26.4 (25.6 to 27.6)	32.3 (31.1 to 34.3)	<0.0001
WC (cm)	79.0 (74.0 to 85.0)	71.0 (68.0 to 76.0)	78.0 (74.0 to 82.0)	90.0 (82.0 to 96.0)	102.0 (96.0 to 110.0)	<0.0001
SBP (mmHg)	113.6 (104.3 to 125.3)	110.3 (101.0 o to 124.0)	113.5 (104.3 to 124.5)	115.0 (105.4 to 127.2)	118.0 (107.3 to 132.2)	0.0001
DBP (mmHg)	73.5 (67.0 to 81.0)	72.0 (65.3 to 79.0)	73.0 (67.0 to 80.0)	75.1 (69.0 to 84.1)	79.4 (72.2 to 87.3)	<0.0001
Pulse rate (beats/min)	75.6 (68.6 to 84.0)	79.0 (69.0 to 86.0)	75.3 (68.3 to 83.6)	76.0 (69.3 to 84.0)	78.0 (71.3 to 85.8)	0.005
Glycaemia (mg/dl)	96.0 (85.0 to 105.0)	96.0 (86.0 to 108.0)	94.5 (84.0 to 104.0)	98.0 (88.0 to 107.0)	100.0 (92.0 to 112.0)	<0.0001
TC (mg/dl)	152.0 (138.0 to 170.0)	151.5 (138.0 to 166.5)	150.0 (136.0 to 169.0)	154.5 (138.0 to 178.5)	161.5 (145.0 to 187.5)	0.0001
HDL-c (mg/dl)	42.0 (38.0 to 48.0)	42.0 (37.0 to 47.0)	42.0 (38.0 to 47.0)	42.0 (38.0 to 49.0)	44.0 (40.0 to 50.0)	0.006
LDL-c (mg/dl)	91.0 (76.0 to 106.0)	93.0 (75.0 to 105.0)	90.0 (76.0 to 105.0)	93.0 (77.0 to 109.0)	93.0 (82.0 to 114.0)	0.002
TG (mg/dl)	98.0 (74.0 to 124.0)	93.5 (72.5 to 126.5)	96.0 (74.0 to 123.0)	99.5 (76.5 to 124.5)	109.5 (76.5 to 136.5)	0.03
Usual physical activity						
None	7.7	8.0	7.2	9.6	10.1	0.03
Moderate	77.2	73.9	76.7	79.1	82.6	
Intense	15.1	18.2	16.1	11.3	7.2	
Intense professional activity						
Yes	49.8	41.6	51.4	48.2	34.2	0.01
Instruction level						
Illiterate/primary	56.4	48.2	56.2	61.7	50.7	0.01
Secondary	41.7	50.2	41.7	36.7	47.8	
University	1.9	1.1	2.1	1.7	1.4	

*BMI* body mass index, *WC*waist circumference, *SBP* systolic blood pressure, *DBP* diastolic blood pressure, *TC* total cholesterol, *HDL-C* high density lipoprotein cholesterol, *LDL-C* low density lipoprotein cholesterol, *TG* triglycerides

In the entire study population at the end of the second cycle, the median interquartile range (IQR) age, BMI, and WC were 34.0 (22.0 to 50.0) years, 22.2 (20.5 to 24.1) kg/m^2^, and 79.0 (74.0 to 85.0) cm, respectively. In the cohort, 77 % had a moderate habitual physical activity, 15.3 % participated in an intense habitual physical activity, and 50.2 % an intense professional activity. Only 7.7 % of the study population was inactive.

Compared to the subgroup of subjects with normal weight, subgroups of obese and malnourished patients had significantly higher median age and blood glucose (*p* <0.001). However, WC, SBP, DBP, Total cholesterol (TC), low-density lipoprotein cholesterol (LDL-C), and TG significantly increased with BMI (*p* <0.05). Finally, the level of physical activity was inversely correlated with BMI (*p* <0.05).

### Dynamic between body mass index and waist circumference

Trends between BMI and WC of the 2062 included subjects since the beginning of the study are noted in Table 2. During the follow-up period (median = 2 years), BMI and WC significantly increased by +0.6 kg/m^2^ and +2.0 cm, respectively. Similarly, the prevalence of overweight individuals and abdominal obesity significantly increased by 23 % (from 11.0 to 14.3 %) and 42.8 % (from 7 to 10.0 %) (*p* <0.0001), respectively. Conversely, the increased prevalence of general obesity (BMI > 30Kg/m^2^) of 7.8 % (3.8 to 4 %) was not significant (*p* = 0.53).

**Table 2:** Trends of risk factors in the study population

	Baseline (*n* = 2.062)	Follow-up (*n* = 2.062)	Difference	*p*
Period (months)	0	24 (24 to 24)	+24	
Age (years)	31.0 (20.0 to 46.0)	33.0 (22.0 to 48.0)	+2.0 years	<0.0001
BMI (Kg/m^2^)	21.7 (20.1 to 23.5)	22.3 (20.8 to 24.2)	+0.6 Kg/m^2^	<0.0001
WC (cm)	77.0 (72.0 to 83.0)	79.0 (74.0 to 85.0)	+2.0 cm	<0.0001
SBP (mmHg)	112.5 (102.5 to 124.0)	114.0 (104.5 to 125.0)	+1.5 mmHg	<0.0001
DBP (mmHg)	72.5 (66.0 to 79.0)	73.5 (67.0 to 80.5)	+1.0 mmHg	<0.0001
Cardiovascular risk factors				
Overweight (%)	11.0	14.3	+23.0 %	<0.0001
Obesity (%)	3.8	4.1	+7.8 %	0.53
Abdominal obesity (%)	7.0	10.0	+42.8 %	<0.0001
AHT (%)	15.0	20.4	+36.0 %	<0.0001

*BMI* body mass index, *WC* waist circumference, *SBP* systolic blood pressure, *DBP* diastolic blood pressure, *AHT* arterial hypertension

### Impact of obesity

Table 3 shows the hazard ratio for the incidence of obesity. During the 4105 person-years observation period, the incidence of obesity was 535/100,000 person-year. The incidence of obesity was significantly higher in subjects between the ages of 40 and 59 (1028/100,000 person-years) compared to subjects <40 years (374/100,000 person-years) and subjects ≥ 60 years (279/100,000 person-years) (*p* <0.0001).

**Table 3:** Hazard ratio for incidence of Obesity

	Number of participants without obesity at start	number who developed obesity	Person-year	/100.000 Person-year	HR (95 % CI)	*p*
	1.842	22	4.105	535	-	-
Gender						
Male	805	8	1.800	444	1	
Female	1.037	14	2.302	608	1.41 (0.59 to 3.35)	0.43
Age (years)						
< 40	1.203	10	2.673	374	1	
40–59	473	11	1.070	1028	2.65 (1.13 to 6.22)	0.02
≥ 60	166	1	358	279	0.79 (0.10 to 6.17)	0.83
Physical activity						
Inactivity	136	2	317	630	1	
Moderate	1.420	19	3.135	606	1.05 (0.24 to 4.94)	0.94
Intense	286	1	647	154	0.26 (0.02 to 2.89)	0.27

### Diabetes mellitus and obesity

Table 4 shows the odds ratio of diabetes mellitus by supposed risk factors. In the overall study population, the prevalence of diabetes mellitus was 2.8 %. Among diabetics, only 9.8 % were obese and 26.9 % had a large WC. In the overall study population, the prevalence of DM increased with WC from 1.6 % in the subgroup in the first WC tertile to 4.0 % in the subgroup of the third WC tertile (*p* = 0.003). The prevalence of DM described a U-shaped curve in terms of BMI with low prevalence in the subgroup with normal weight (2.1 %) and a relatively high prevalence in malnourished people (2.4 %) and a significantly higher prevalence in overweight (4.3 %) and obese (7.4 %) subjects (*p* = 0.0005).

**Table 4:** Odd ratio of diabetes mellitus by supposed risk factors

	Univariate	Model 1	Model 2
Age > 40 years	2.21 (1.44 to 3.38)	-	-
High WC	4.09 (2.44 to 6.87)	3.48 (2.04 to 5.93)	-
TG > 150 mg/dl	1.52 (0.79 to 2.91)	-	-
AHT	3.26 (2.12 to 5.02)	2.79 (1.76 to 4.41)	1.93 (1.14 to 3.24)
BMI category			
Normal	1	1	1
Undernutrition	1.14 (0.45 to 2.90)	1.04 (0.40 to 2.64)	1.06 (0.41 to 2.70)
Overweight	2.17 (1.26 to 3.72)	1.99 (1.14 to 3.46)	1.24 (0.64 to 2.42)
Obesity	3.69 (1.70 to 8.00)	3.17 (1.45 to 6.93)	1.38 (0.52 to 3.66)

Model 1. After adjusted for age

Model 2. After adjusted for age and WC

In the logistic regression, ANOVA, however, the association between BMI and DM was no longer significant after adjustment for age and WC. Only blood pressure showed an independent effect on the probability of DM (adjusted OR = 1.93; *p* = 0.01).

### Metabolic syndrome

In the entire group, the prevalence of MS was 7.2 %. The prevalence of MS was higher in the group with diabetes than in the group without diabetes (40.0 % vs 6.3 %, respectively; *p* <0.0001) and increased with BMI from 6.3 % in malnourished to 38.3 % in obese participants (*p* <0.0001).

In the logistic regression, multivariate analysis, being overweight (adjusted OR = 4.1; *p* = 0.0002) or obese (adjusted OR = 11.8; *p* <0.0001) were factors that were independently associated with MS risk after adjusting for age, female gender, and DM.

## Discussion

This study confirms the relationship between DM and abdominal obesity but not with general obesity. However, the prevalence of DM (2.8 %) was low. Similarly, the prevalence of obesity (9.8 %) and MS (40.0 %) of the group with DM was low. The study also notes a significant increase in BMI (+0.6 kg/m^2^), WC (+2.0 cm), and abdominal obesity (+42.8 %) in this rural population during a median 24-month follow-up period. This group was also more overweight (+ 23.0 %), the incidence of obesity was 5.35/1000 person-year and higher in people between the ages of 40 and 59.

The results of this study are in line with the literature, which reports a low prevalence of DM in rural areas of sub-Saharan Africa. Other authors documented the prevalence of DM at 2.8 % in rural Angola [21] and 2.2 % in rural Nigeria [22]. A low prevalence of DM was also found in Kaziba (2.9 %), a rural locality within the same province as this study [10].

In addition, the prevalence of being overweight/obese was significantly lower in rural areas than in urban areas of sub-Saharan Africa [10, 23]. This shows that people in rural areas of sub-Saharan Africa mostly keep their traditional active lifestyle. However, economic, nutritional, and epidemiological transitions seems to be faster in rural areas of developed countries [24] and some North African regions [25].

Analyses of BMI and WC trends in the study population showed both an increase in the prevalence of being overweight and abdominal obesity during the study period. The increase in BMI was 0.6 kg/m^2^ during the median 2-year follow-up period. This increase is significantly higher compared to the global increase in BMI, which is estimated at 0.4 kg/m^2^ every 10 years for men and 0.5 kg/m^2^ every 10 years for women [26]. This observation may suggest a rapid increase in “diabesity” in the near future in this rural area. This would be in line with the DM global prevalence projection of 98.1 % in the number of diabetics in Africa by 2030 [2]. In addition, the urbanization rate is fast on this continent and will reach 47.7 % in 2030 compared with 39.6 % in 2011 [27].

There was a relatively high prevalence of DM in the malnourished subgroup compared to subjects with a normal weight. In addition, the frequency of obesity (BMI > 30 kg/m^2^) in the group with DM was very low (9.8 %). This still confirms the high frequency of atypical diabetes in this rural area and is consistent with what is reported in other rural African populations [10, 21, 28].

The mechanisms of this atypical form of DM are not well-known, but several hypotheses are proposed including infection with HHV-8 [29] and hyperferritinemia [30]. Also in this study, the association between DM and BMI categories disappeared after adjustment for WC. In addition, only age, abdominal obesity, and AHT remained associated with diabetes mellitus risk after adjustment for all confounders. Thus, the higher age of the malnourished subjects compared to subjects with normal weight may account for a relatively high prevalence of DM in malnourished. Further, our observation confirms the major role of perivisceral fat accumulation in the pathogenesis of DM in this rural area despite a low prevalence of obesity.

The literature reports an association between DM and abdominal obesity and not between DM and obesity as defined by BMI in African rural areas [22]. The underlying contributor of “diabesity” may be the insulin resistance of which the best clinical markers are abdominal obesity and high blood pressure and not obesity defined by BMI. Theoretically, this observation suggests revising the definition of “diabesity” and to consider the association between diabetes mellitus and abdominal obesity and not between diabetes mellitus and obesity defined by BMI. Using this assumption of “diabesity”, 26.9 %, not 9.8 %, of this study’s cohort would be diabetic. However, even after this adjustment, the prevalence of “diabesity” would still be low for this rural area.

The main limitation of this study is the definition of DM. Being diabetic was based only on fasting blood glucose or taking medication. This may have underestimated the prevalence of diabetes mellitus. The addition of an oral glucose tolerance test and/or glycated hemoglobin HbA1c would have been helpful. In addition, blood glucose and lipid measurements were only done in the third year of the study, which does not help to estimate the incidence of DM and MS.

## Conclusion

This study reports a low prevalence of diabetes mellitus and obesity for people living in the rural area of Katana. The study also shows an association between diabetes mellitus and abdominal obesity but not with general obesity. In addition, the rapid increase in abdominal obesity prevalence in this rural area population within the follow-up period supports the need for urgent lifestyle preventive measures in this area.

## Abbreviations

AHT: Arterial hypertension
BMI: Body mass index
DBP: Diastolic blood pressure
DM: Diabetes mellitus
HDL-C: High-density lipoprotein cholesterol
LDL-C: Low-density lipoprotein cholesterol
MS: Metabolic syndrome
SBP: Systolic blood pressure
TC: Total cholesterol
TG: Triglycerides
WC: Waist circumference

## References

[1] IDF (2013). Diabetes atlas.

[2] Wild S, Roglic G, Green A, Sicree R, King H (2004). Global prevalence of diabetes: estimates for the year 2000 and projections for 2030. Diabetes Care.

[3] World Health Organization (1994). Prevention of diabetes mellitus. Technical Report Series no. 844.

[4] Bray GA (2003). Risks of obesity. Endocrinol Metab Clin North Am.

[5] Hossain P, Kawar B, El Nahas M (2007). Obesity and diabetes in the developing world–a growing challenge. N Engl J Med..

[6] Kalra S (2013). Diabesity. J Pak Med Assoc.

[7] Alberti G, Eckel R, Grundy S, Zimmet P, Cleeman J, Donato K (2009). Harmonizing the Metabolic Syndrome. A Joint Interim Statement of the I.D.F Task Force on Epidemiology and Prevention; NHL and Blood Institute; AHA; WHF; IAS; and IA for the Study of Obesity. Circulation.

[8] Tuei VC, Maiyoh GK, Ha CE (2010). Type 2 diabetes mellitus and obesity in sub-Saharan Africa. Diabetes Metab Res Rev.

[9] Dalal S, Beunza JJ, Volmink J, Adebamowo C, Bajunirwe F, Njelekela M (2011). Non-communicable diseases in sub-Saharan Africa: what we know now. Int J Epidemiol.

[10] Katchunga P, Masumbuko B, Belma M, Kashongwe Munogolo Z, Hermans MP, M’buyamba-Kabangu JR (2012). Age and living in an urban environment are major determinants of diabetes among South Kivu Congolese adults. Diabetes Metab.

[11] Mapatano MA, Muyer MC, Buntinx F, De Clerck M, Okitolonda W, Bieleli IA (2007). Obesity in diabetic patients in Kinshasa. Democratic Republic of Congo. Acta Clin Belg.

[12] Mauvais-Jarvis F, Sobngwi E, Porcher R, Riveline JP, Kevorkian JP, Vaisse C (2004). Ketosis-prone type 2 diabetes in patients of sub-Saharan African origin: clinical pathophysiology and natural history of beta-cell dysfunction and insulin resistance. Diabetes.

[13] Katchunga P, Hermans MP, Manwa B, Lepira F, Kashongwe Z (2010). Hypertension, insulin resistance and chronic kidney disease in type 2 diabetes patients from South Kivu, DR Congo. Nephrol Ther.

[14] Katchunga PB, Twagirumukiza M, Kluyskens Y, Kaishusha D, Baguma M, Bapolisi A (2015). Blood pressure in the Congolese adult population of South Kivu, Democratic Republic of Congo: Preliminary results from the Bukavu Observ Cohort Study. Rev Epidemiol Sante Publique.

[15] World Health Organization. The STEPS instrument and support materials. Available at: http://www.who.int/chp/steps/instrument/en/index.html. Accessed 6 Aug 2011.

[16] Mancia G, Fagard R, Narkiewicz K, Redón J, Zanchetti A, Böhm M (2013). ESH/ESC Guidelines for the management of arterial hypertension: the Task Force for the management of arterial hypertension of the European Society of Hypertension (ESH) and of the European Society of Cardiology (ESC). J Hypertens.

[17] Alberti KG, Zimmet P, Shaw J, IDF Epidemiology Task Force Consensus Group (2005). The metabolic syndrome: a new worldwide definition. Lancet.

[18] Katchunga BP, Hermans MP, Bamuleke AB, Katoto CP, Kabinda MJ (2013). Relationship between waist circumference, visceral fat and metabolic syndrome in a Congolese community: Further investigations are still very necessary. PAMJ.

[19] Longo-Mbenza B, Kasiam Lasi On’kin JB, Nge Okwe A, Kangola Kabangu N (2011). The metabolic syndrome in a Congolese population and its implications for metabolic syndrome definitions. Diabetes Metab Syndr.

[20] Sumner AE, Sen S, Ricks M, Frempong BA, Sebring NG, Kushner H (2008). Determining the waist circumference in African Americans which best predicts insulin resistance. Obesity.

[21] Evaristo-Neto AD, Foss-Freitas MC, Foss MC (2010). Prevalence of diabetes mellitus and impaired glucose tolerance in a rural community of Angola. Diabetol Metab Syndr.

[22] Alikor CA, Emem-Chioma PC (2015). Epidemiology of diabetes and impaired fasting glucose in a rural community of Nigerian Niger Delta region. Niger J Med.

[23] Ziraba AK, Fotso JC, Ochako R (2009). Overweight and obesity in urban Africa: A problem of the rich or the poor?. BMC Public Health.

[24] Melidonis AM, Tournis SM, Kompoti MK, Lentzas IL, Roussou VR, Iraklianou SL (2006). Increased prevalence of diabetes mellitus in a rural Greek population. Rural Remote Health.

[25] Toselli S, Gualdi-Russo E, Boulos DN, Anwar WA, Lakhoua C, Jaouadi I (2014). Prevalence of overweight and obesity in adults from North Africa. Eur J Public Health.

[26] Finucane MM, Stevens GA, Cowan MJ, Danaei G, Lin JK, Paciorek CJ (2011). National, regional, and global trends in body-mass index since 1980: systematic analysis of health examination surveys and epidemiological studies with 960 country-years and 9 · 1 million participants. Lancet.

[27] World Urbanization Prospects (2012). The 2011 Revision, United Nations Department of Economic and Social Affairs.

[28] Hall V, Thomsen RW, Henriksen O, Lohse N (2011). Diabetes in Sub Saharan Africa 1999-2011: epidemiology and public health implications. A systematic review. BMC Public Health.

[29] Sobngwi E, Choukem SP, Agbalika F, Blondeau B, Fetita LS, Lebbe C (2008). Ketosis-prone type 2 diabetes mellitus and human herpesvirus 8 infection in sub-saharan Africans. JAMA.

[30] Katchunga PB, Baguma M, M’buyamba-Kabangu JR, Philippé J, Hermans MP, Delanghe J (2013). Ferroportin Q248H mutation, hyperferritinemia and atypical type 2 diabetes mellitus in South Kivu. Diabetes Metab Syndr.

